# Diurnal and seasonal variation of particle and dissolved organic matter release by the coral *Acropora tenuis*

**DOI:** 10.7717/peerj.5728

**Published:** 2018-11-07

**Authors:** Haruko Kurihara, Nao Ikeda, Yu Umezawa

**Affiliations:** 1 Faculty of Science, University of the Ryukyus, Okinawa, Japan; 2 Department of Environmental Science on Biosphere, Tokyo University of Agriculture and Technology, Fuchu city, Tokyo, Japan

**Keywords:** Mucus, Coral, *Acropora tenuis*, Dissolved organic matter, Particle organic matter

## Abstract

Release rates of particulate organic carbon and nitrogen (POC and PON) and dissolved organic carbon (DOC) from the scleractinian coral *Acropora tenuis* were measured during the day and night in summer and winter seasons. Physiological parameters including calcification, photosynthesis and respiration rates were also measured simultaneously. The release rate of both POC and DOC was significantly higher in summer compared to winter and higher during the day compared to the night. The daily release rate of total organic carbon (POC + DOC) was 1,094 and 219 μmol C cm^−2^ d^−1^ for summer and winter, respectively, being 4.9 times higher in summer. The POC:PON ratios of the particulate organic matter released during daytime in both seasons (summer: 12.8 ± 5.7, winter: 12.0 ± 4.1) were significantly higher than those during nighttime (summer: 6.1 ± 2.5, winter: 2.2 ± 1.8). The DOC:POC ratio was 0.5 ± 0.03 during summer and 0.32 ± 0.98 during winter, suggesting higher mucus release in particulate form. Daily net production was estimated to be 199 and 158 μg C cm^−2^d^−1^ for summer and winter, respectively, with the amount of carbon released as mucus accounting for 6.5% and 1.6% of the net carbon fixation, respectively. The study reveals diurnal and seasonal changes in the quantity and quality of mucus released from this coral species. Since coral mucus is used as a food source by reef macro-organisms, and can also serve as an energy source for micro-organisms, the observed changes in mucus release rates are expected to influence the seasonal dynamics of organic carbon and nitrogen cycling over coral reefs.

## Introduction

Corals continuously release a large amount of organic matter in the form of mucus to the surrounding water (Davies, 1984; Crossland, 1984). Since coral reefs are an oligotrophic ecosystem, mucus has been suggested to serve as an essential source of nutrients, helping to sustain the high productivity and biodiversity of these systems (Marshall, 1986). Mucus can be released as particulate organic matter (POM), or as dissolved organic matter (DOM), and is defined as a mixture of organic compounds containing glycoproteins such as mucins, carbohydrate and lipids (Mitchell & Chet, 1975; Wild et al., 2010; Bythell & Wild, 2011). Several functions of mucus have been proposed for corals and have been reviewed in detail by Brown & Bythell (2005); the functions include defenses against desiccation (Krupp, 1984), sedimentation (Schuhmacher, 1977; Rogers, 1990), UV damage (Drollet et al., 1997), pathogenesis (Ritchle, 2006) other pollutants such as oil and heavy metals (Mitchell & Chet, 1975) and physical damage (Meesters, Moordeloos & Bak, 1994). The mucus released into the water is also known to serve as an important organic carbon source for coral reef organisms (Marshall, 1965). For instance, mucus released as POM can be used as a food source by other reef organisms, including fishes and macro-benthos (Benson & Muscatine, 1974; Mayer & Wild, 2010; Naumann et al., 2010a). Mucus can also trap organic matter in the water column, and be consumed by heterotrophic organisms, preventing the loss of organic matter from the reef ecosystem (Wild et al., 2004). Coles & Strathmann (1973) measured the C:N ratio of the mucus of the coral *Porites* sp. and *Acropora* sp. corals, and proposed that the released mucus becomes enriched with nitrogen once in the seawater, and can thus be an essential nutrient source for other organisms. More recently, it has been suggested that mucus released as DOM could be used as a source of nutrients and organic matter for micro-organisms such as bacteria (Haas et al., 2011; Nakajima et al., 2009, 2015, 2017; Tanaka & Nakajima, 2018). Taking all these functions of coral mucus into account, precise evaluation of the productivity of coral mucus is essential to understand energy flow and biogeochemical cycling over reef ecosystems.

The metabolic demand of corals for mucus production has been studied since Cooksey & Cooksey (1972) indicated that half of the photosynthetic products fixed as carbon by the endosymbiotic dinoflagellates can be lost from the coral within one day. Crossland, Barnes & Borowitzka (1980) also estimated that mucus formation accounts for as much as 40% of the net production by coral. Similarly, for the coral *Poccilopora eydouxi,* it was estimated that about 48% of the fixed energy by photosynthesis is lost as mucus, 51% is used in respiration and 0.9% for growth (Davies, 1984). Meanwhile, Muscatine et al. (1984) reported that the light-adapted coral *Stylophora pistillata* released only 6% fixed energy, while shade-adapted coral released almost half of the carbon it fixed. However, most of these studies evaluated mucus formation in the context of energy budgets, and quantitative studies that have directly measured directly the amount of mucus production in the form of POM and DOM by corals are still limited (Brown & Bythell, 2005).

Crossland (1987) measured the daily dissolved organic carbon (DOC) mucus released by the corals *Acropora variabilis* and *S. pistillata* using a pump system chamber in the field for 48 h, and it was estimated that *A. variabilis* and *S. pistillata* produced 16.2 and 27.5 μg C cm^−2^ d^−1^ DOC-lipid which represent about 8% and 10–20% of the daily fixed carbon, respectively. These authors also demonstrated that the amount of mucus released by corals showed diurnal variation and was affected by light irradiance. Wild, Woyt & Huettel (2005) quantified mucus production by the corals *A. millepora* and *A. aspera* in beakers and in situ chambers, demonstrating that air exposure increases mucus release rate by about two times. Tanaka et al. (2008) conducted simultaneous measurement of both net production of POM and DOM for two coral species, *A. pulchra* and *Porites cylindrica* using a chamber method and demonstrated that both corals produced higher amounts of POM compared to DOM, and that DOC production accounted for less than 10% of newly synthesized organic carbon. Additionally, in another study Tanaka, Ogawa & Miyajima (2010) demonstrated that nutrient enrichment decreases the dissolved organic nitrogen (DON) release rate in the coral *Montipora digitata*, suggesting that nutrient concentration can also affect mucus release. All these studies together suggest that mucus release rates are dependent on coral metabolic activity and can change with environmental conditions.

Temperature, light intensity and nutrient concentrations are known to strongly affect coral metabolism and calcification (Coles & Jokiel, 1977; Crossland, 1981, 1984; Marshall & Clode, 2004). Studies by Kinsey (1977) and Smith (1981) indicated that coral community calcification and organic carbon production in high latitude coral reefs show strong seasonality in correlation with light and temperature changes. Other studies also indicated that coral tissue biomass, symbiotic dinoflagellate density and chlorophyll *a* concentrations can change between seasons (Brown et al., 1999; Fitt et al., 2000). Therefore, it is hypothesized that the mucus production rate of high latitude corals, where the environmental conditions may change drastically with the seasons, will markedly change according to concomitant change in coral physiology. Naumann et al. (2010b) first evaluated the seasonal change of mucus amount released by six coral species and indicated that the amount of particulate organic carbon and nitrogen (POC and PON) were affected by ambient conditions such as water temperature and light intensity. However, they also indicated that out of the six studied corals, only *Acropora* showed significant seasonal differences of POC release. Additionally, Levas et al. (2016) reported that the DOC release of coral *Porites divaricata* showed seasonal differences, and DOC uptake was observed in late summer while DOC release was observed in spring/early summer. However, until now there have still only been a limited number of studies that have evaluated the seasonal changes of mucus production by corals. Additionally, even though it is hypothesized that the amount of mucus changes with coral physiology, there have been even less studies evaluating coral metabolism and mucus release rates at the same time.

In this study, we aimed to quantify the amount of mucus released as POM (here measured as POC and PON) and DOM (here measured only as DOC) during the day and night in two different seasons (summer and winter), focusing on a coral living in the high-latitude sub-tropical reefs in Okinawa. The coral *A. tenuis,* which a common species in Pacific coral reef ecosystems (Veron, 2000), was used to obtain simultaneous measurements of net photosynthesis, respiration, light and dark calcification rates and mucus release rates in order to clarify the relative contribution of mucus production to the coral’s metabolism.

## Materials and Methods

### Study site and coral sampling

Three colonies of the coral *A. tenuis* were sampled on July 30, 2013 and November 15, 2013 from inshore patch reefs (ca. two to five m depth) in front of the Sesoko Station, University of the Ryukyus, Okinawa Island, Japan (26°38′11.64″N, 127°51′57.08″E). All corals were collected under permission from Okinawa Prefecture (#24–65). Four fragments with approximately similar surface area (51 ± 12 cm^2^) were made from each of the three colonies. Each fragment was glued on plastic screws and acclimatized for around one month in an outdoor tank (95 × 60 × 17 cm, volume = 295 L) continuously supplied with seawater pumped from four to five m depth in front of the station. Seawater temperatures in the outdoor tank were monitored every 30 min using a data logger (Hobo U22-001; Onset Computer Corp., Bourne, MA, USA) during the coral acclimation; temperature averaged 28.9 ± 0.6 °C and 22.7 ± 1.1 °C during the summer and winter, respectively. Light intensity was also monitored every 10 min using a data logger (JFE-Advantec Co., Ltd., Kobe, Japan) that was placed in the outdoor tank, with mean daily integrated intensity ranging from 315 to 643 μmol photons m^−2^ s^−1^ (average: 499) and 73–397 μmol photons m^−2^ s^−1^ (average: 220) during summer and winter, respectively.

### Coral incubation

#### Experimental set-up

To quantify mucus release rates and the physiological state of the corals, bottle incubations were conducted in summer (September 7–8, 2013) and winter (December 27–28, 2013) under light and dark conditions. After acclimation, corals were transferred from the outdoor to an indoor tank for bottle incubations without air exposure. After 1 day of acclimation in the indoor tank, each coral fragment was incubated individually within acid-rinsed glass bottles (volume 800 ml) with a transparent screw cap with two small holes. Bottles containing corals (*n* = 12) and control bottles without coral (*n* = 5) were placed in a water bath, and new running filtered (one-μm φ) seawater was supplied into the bottom of the bottles (250 ml min^−1^) through the tubes connected into the holes in the screw cap for another day. Running seawater was also continuously supplied to the water bath to control temperature (summer: 28.8 ± 0.6 °C and winter: 20.1 ± 0.6 °C). Two metal halide lumps (summer: 394 μmol photons m^−2^ s^−1^ and winter: 351 μmol photons m^−2^ s^−1^) were set above the bottles to control the light intensity. On the first day, a 3 h incubation was conducted to quantify the amount of mucus released as dissolved organic C (DOC) and particulate organic C (POC) and N (PON) by the corals under light conditions (11:00–14:00 h). At night (20:00–23:00) on the same day, dark calcification and respiration were measured by the alkalinity-dissolved inorganic carbon (DIC) anomaly technique (Smith, 1973; Smith & Key, 1975; Chisholm & Gattuso, 1991; Langdon & Atkinson, 2005). The following day, a 3 h incubation was conducted using the same corals to measure the light calcification rate and photosynthesis rate under light conditions, and then the amounts of DOC, POC, PON released were measured under dark conditions.

#### Sample collection

During daytime on the first day, just before starting the incubation, seawater for initial DOC measurement was sampled from all control (*n* = 5) and coral bottles (*n* =12) using acid-rinsed glass syringes. After rinsing the filter two times, seawater was filtered into pre-combusted 10 ml amber glass vials in duplicate. These samples were kept frozen at −20 °C until analyses. Thereafter, seawater within control bottles (*n* = 5) was sampled using sterile plastic syringes for measurement of the initial dissolved inorganic nitrogen (DIN) and dissolved inorganic phosphorus (DIP) concentration. 10 ml seawater was filtered with pre-combusted (450 °C; 4 h) GF/F filter and after rinsing the filter and plastic tubes two times, was collected into the tubes in duplicate and stored in a deep freezer until analyses. Seawater for DOC measurement was also filtered with pre-combusted (450 °C; 4 h) GF/F filter using glass syringe pre-rinsed with acid after rinsing the filter and syringe for two times. Filtered seawater was transferred to pre-combusted (450 °C; 4 h) ampoules, sealed and stored at deep freezer until analysis. The volume of the remaining seawater in the control bottles was measured and filtered using pre-combusted (450 °C, 4 h) and pre-weighted GF/F filters for the initial POC and PON analyses. All filters were dried in an oven at 60 °C overnight, treated with 0.5N HCl, and then dried and weighed again. After all seawater sampling, bottles were refilled with new filtered seawater and then the running seawater was stopped by closing the holes. To minimize the stress by incubating corals within a closed chamber, seawater within the bottle was continuously stirred using a magnetic stirrer (mean speed: 3.4 cm s^−1^) during the 3 h incubation, following the method used by Tanaka et al. (2008). Additionally, coral size was limited to less than 10% of the total seawater volume within the chamber. After 3 h incubation, corals were immediately taken out of the bottles and transferred within the water bath, with water samples for DIN, DIP, DOC and POC analyses taken immediately from all bottles in the same manner as described above. After seawater sampling, each bottle was refilled with filtered seawater, and each coral fragment placed back into its bottle and maintained under running fresh seawater until dark incubation.

During the nighttime of the first day, just before starting the dark incubation, seawater samples for the initial DIC and total alkalinity (TA) measurement were collected from control bottles (*n* = 5) by siphoning into 100 ml pre-combusted glass vials. Sampled seawater was immediately fixed with 100 μl saturated mercuric chloride and kept until measurement. After seawater sampling, all chambers were re-filled with new flowing seawater without head space, and running seawater was stopped and closed for the 3 h incubation. All experimental bottles were mixed with a magnetic stirrer during culture, and after incubation, 100 ml of seawater was sampled from all bottles (*n* = 17) and fixed with 100 μl saturated mercuric chloride for DIC and TA measurement. After sampling, all bottles were refilled with seawater and incubated under running seawater until the next day.

On the second day, sampling for DIC and TA measurements during the daytime, and for DOC, POC, PON, DIN and DIP during the nighttime, were conducted in the same manner as on the first day. After finishing all incubations, the volume of the seawater within each bottle was recorded and the surface area of each coral measured using the aluminum foil technique (Marsh, 1970).

#### Sample *analyses*

DIN (NO_3_^−^+ NO_2_^−^ and NH_4_^+^) and DIP (PO_4_^3−^) was measured using an AACS III (BLTEC). To draw calibration lines, standard solutions were prepared using KNO_3_ for NO_3_^−^, NaNO_2_ for NO_2_^−^, (NH_4_)_2_SO_4_ for NH_4_^+^, and KH_2_PO_4_ for PO_4_^3−^. DOC was analyzed using TOC-L (Shimadzu, Kyoto, Japan), and calibrated with a standard curve of glucose diluted with Milli Q water (0, 0.5, 1, 2, 3 mg/L). All standard solutions were analyzed by the same procedure as the samples. Milli-Q (18.2 MΩ) was used as a blank (average 0.01 mgC/L). Samples were acidified with 9N H_2_SO_4_ before measurement to completely remove inorganic carbon species, and typical precision was 0.02 mgC (SD).

Particulate organic carbon and nitrogen was measured by a CN elemental analyzer (Sumigraph NC-22A). Acetanilide was used as a standard material for calibration. A blank filter was prepared using Milli-Q water in the same manner as the samples and sample values were obtained by subtracting the blank value from the measured value.

Dissolved inorganic carbon and TA were measured using closed cell titration (ATT-05; Kimoto, Tokyo, Japan) and 0.1N HCl. The accuracy and precision of DIC and TA determinations was evaluated by analyzing reference material Batch AG from KANSO TECHNOS, JAPAN (TA = 2295 ± 0.55 μmol Kg^−1^, DIC = 2032.8 ± 0.72 μmol Kg^−1^), which was traced with certified reference material (CRM, Batch 95, from A. Dickson Laboratory).

### Data calculation

The net release rate of DOC by each of the 12 coral colonies (NDOC: nmol C cm^−2^ h^−1^) was calculated using the following equation:

$${\rm{NDOC }} = {\rm{ }}(\Delta {\rm{DOC}}-\Delta {\rm{DOCc}}){\rm{ }}V{\rm{ }}{S^{-1}}{t^{-1}}$$

where ΔDOC is the DOC (nmol C L^−1^) change during each of the 12 coral bottle incubations, and ΔDOCc is the average DOC change during incubation of the five control bottles, *V* (L) is the seawater volume within each bottle, *S* is coral surface area (cm^2^) and *t* is the duration of culture (h). For DOC, the initial value in the coral bottles was also evaluated to eliminate the potential for release of DOM at the coral surface at time 0.

The net release rates of POC and PON by the corals (NPOC, NPON: nmol C cm^−2^ h^−1^, nmol N cm^−2^ h^−1^) were calculated using the following equations:

$${\rm{NPOC}}\left({\rm{N}} \right)= {\rm{ }}\Delta {\rm{POC}}\left(N \right){\rm{ }}V{\rm{ }}{S^{-1}}{t^{-1}}$$

where ΔPOC(N) was calculated by subtracting the mean POC(N) (nmol C (N) L^−1^) of control bottles measured at the end of incubation and the POC(N) change in control bottles during the 3 h incubation from each POC(N) measured at the end of incubation of the 12 coral bottles.

Net calcification rates (G: μmol CaCO_3_ cm^−2^ h^−1^) were calculated using the following equation:

$${\rm{Light }}\left({{\rm{Dark}}} \right){\rm{ }}G{\rm{ }} = {\rm{ }}\Delta {\rm{TA V}}{{\rm{D}}_{sw}}{\left({2{\rm{ }}S{\rm{ }}t} \right)^{-1}}$$

where ΔTA is the TA (μmol Kg^−1^) change during the daytime under light or at nighttime under dark conditions, calculated by subtracting the mean TA of the five control bottles measured at the end of the incubation and the TA change in control bottles during the 3 h incubation from each TA measured at the end of incubation of 12 coral bottles, and D_*sw*_ is the seawater density.

Respiration (R: μmol C cm^−2^ h^−1^) and net photosynthesis (P: μmol C cm^−2^ h^−1^) were calculated using the following equation:

$${\rm{nP }}\,or \,{\rm{ }}R{\rm{ }} = {\rm{ }}\Delta {\rm{DIC\, V}}{{\rm{D}}_{sw}}{\left({S\,t} \right)^{-1}}-{\rm{ }}G$$

where ΔDIC is the DIC (μmol Kg^−1^) change during the light or dark incubations calculated by subtracting the mean DIC of the control bottles measured at the end of the incubation and the DIC change in control bottles during the 3 h incubation from each DIC measured at the end of incubation of 12 coral bottles. Gross photosynthesis was calculating by adding net photosynthesis and dark respiration.

### Statistical analyses

Mixed-effect-model ANOVA based on the restricted maximum likelihood (REML) method was performed to evaluate the differences in release rates of mucus (POC, PON and DOC) between seasons (summer and winter) and between day and night time, with seasons and day and night time as fixed effects and colony as a random effect. Differences between day and nighttime and seasons were assessed using Tukey’s honestly significant difference (HSD) multiple comparison tests. Differences in light and dark calcification rate, and net photosynthesis rates between seasons were evaluated using REML with seasons as fixed effects and colony as a random effect. Normality and equal variance were confirmed with the Shapiro-Wilk, Levene’s test and Barlett test. POC, PON, C:N, calcification and gross photosynthesis data were log transformed for normality. Because respiration rate did not meet the assumption of normality, data were analyzed with non-parametric Wilcoxon test. All analyses were performed using JMP (JMP 7; SAS Inc., Cary, NC, USA).

## Results

### Seawater environment

Seawater temperatures and daytime light intensity in summer and winter indoor incubations averaged 28.8 ± 0.6 °C and 394 μmol photons m^−2^ s^−1^, and 20.1 ± 0.6 °C and 351 μmol photons m^−2^ s^−1^, respectively. POC and PON concentrations in the control incubation in summer and winter conditions averaged 10.9 ± 0.2 and 1.9 ± 0.2 μmol C L^−1^ for POC and 1.4 ± 0.05 and 0.2 ± 0.02 μmol N L^−1^ for PON, respectively. DOC concentrations in the control incubation in summer and winter conditions averaged 54.7 ± 1.3 and 49.1 ± 2.0 μmol C L^−1^, respectively. The seawater nutrient concentrations in the control incubation seawater summer and winter averaged 0.18 ± 0.01 and 0.18 ± 0.04 μmol L^−1^ for NO_3_^−^+ NO_2_^−^, 0.07 ± 0.08 and 0.04 ± 0.01 μmol L^−1^ for NH_4_^+^, and 0.04 ± 0.002 and 0.04 ± 0.004 μmol L^−1^ for PO_4_^3−^, respectively.

### Mucus release (POC, PON, DOC)

**Figure 1:**
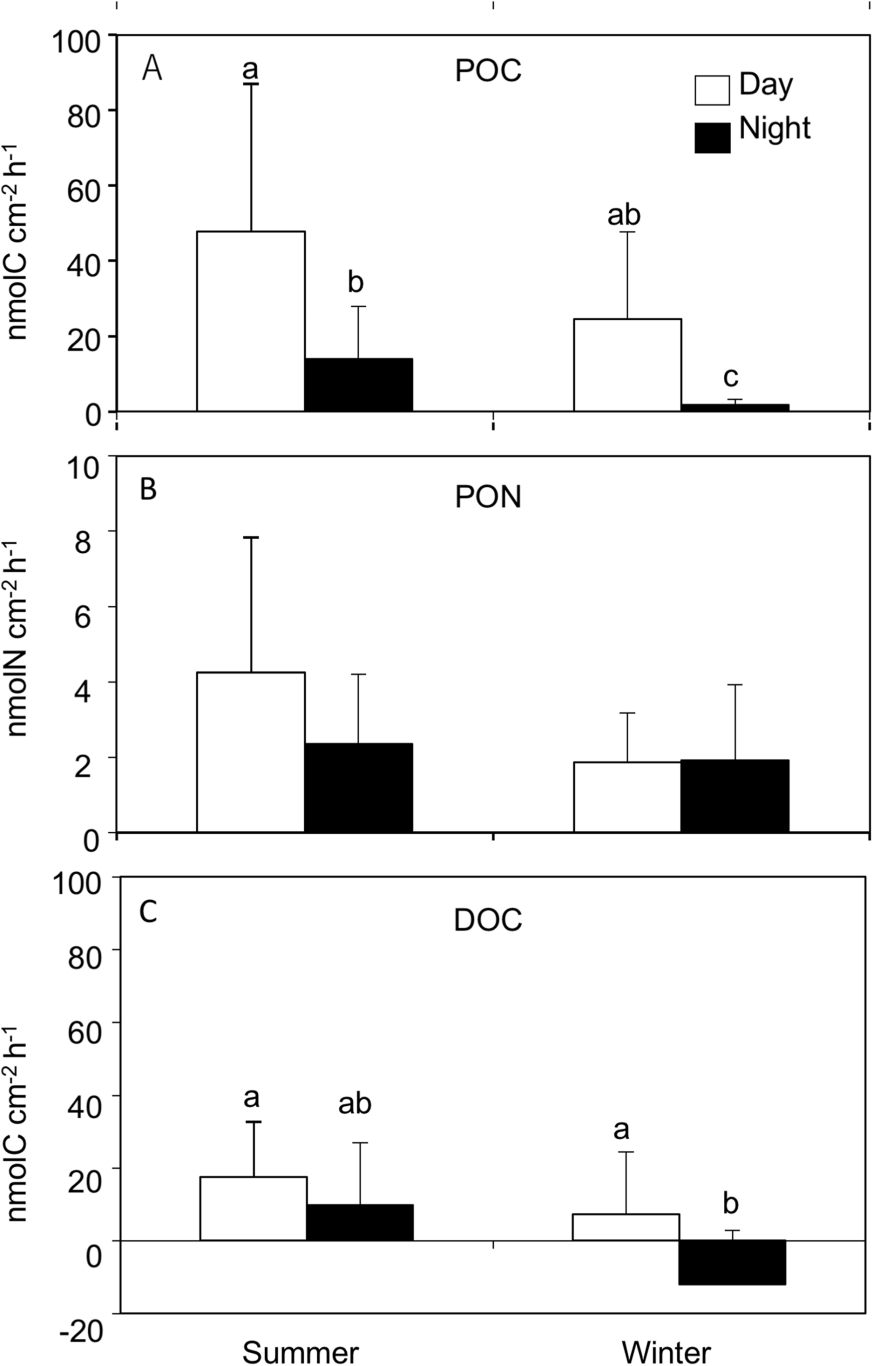
Release rates for (A) particle organic C (POC), (B) particle organic N (PON) and (C) dissolved organic C (DOC) from *Acropora tenuis* during the summer and winter day and night time. Values are mean and SD. (*n* = 12). Bars with different small letters show significant differences among treatments (HSD Tukey–Krammer, *p* < 0.05).

Particulate organic carbon release rates were significantly different between seasons (REML, *F*_(1,4.2)_ = 34.02, *p* = 0.003), and between day and nighttime (REML, *F*_(1,38.3)_ = 59.96, *p* < 0.0001), with an interaction (REML, *F*_(1,38.3)_ = 4.63, *p* = 0.03). The average POC release rates were significantly higher during the day in both summer (47.7 ± 39.2 nmol C cm^−2^ h^−1^) and winter (24.5 ± 23.1 nmol C cm^−2^ h^−1^) compared to the night at summer (13.9 ± 14.0 nmol C cm^−2^ h^−1^) and winter (1.8 ± 1.3 nmol C cm^−2^ h^−1^, Tukey HSD test, *p* < 0.05, Fig. 1; Table 1) respectively. The highest PON release rate was observed during the day in summer (4.2 ± 3.5 nmol N cm^−2^ h^−1^), while the lowest was observed during the day in winter (1.8 ± 1.3 nmol N cm^−2^ h^−1^), however there was no significant difference between seasons (REML, *F*_(1,4.2)_ = 6.73, *p* = 0.05) or day and night (REML, *F*_(1,38.3)_ = 2.34, *p* = 0.13, Fig. 1; Table 1). POC:PON ratios during daytime in both summer (12.8 ± 5.7) and winter (12.0 ± 4.1) were significantly higher than those during the night summer (6.1 ± 2.5) and winter (2.2 ± 1.8), respectively (REML, *F*_(1,38)_ = 11.3, *p* < 0.0001). However, there was no significant difference between seasons (REML, *F*_(1,4)_ = 80.7, *p* = 0.02).

**Table 1:** Summer and winter release rates of particulate (POC, PON) and dissolved organic matter (DOC), and metabolic rate, including calcification (G), net photosynthesis (NP), respiration (R) and gross photosynthesis of the coral *Acropora tenuis*.

		POC (nmol cm^−2^ h^−1^)			PON (nmol cm^−2^ d^−1^)			DOC (nmol cm^−2^ d^−1^)			G (μmol cm^−2^ d^−1^)			NPorR (μmol cm^−2^ d^−1^)			GP (μmol cm^−2^ d^−1^)		
Summer	Day	47.7	±	39.2	4.2	±	3.5	26.2	±	17.4	0.47	±	0.14	1.2	±	0.2	1.3	±	0.2
	Night	13.8	±	14.0	2.3	±	1.8	4.8	±	27.4	0.2	±	0.05	0.1	±	0.1			
Winter	Day	24.5	±	23.0	1.8	±	1.3	−4.1	±	12.7	0.42	±	0.06	1.2	±	0.2	1.3	±	0.1
	Night	1.8	±	1.3	1.9	±	2.0	−17.2	±	21.2	0.16	±	0.06	0.06	±	0.05			

Dissolved organic carbon release rates were significantly different between seasons (REML, *F*_(1,4.0)_ = 14.52, *p* = 0.01), and between day and night (REML, *F*_(1,38.9)_ = 9.95, *p* = 0.003), without an interaction (REML, *F*_(1,38.9)_ = 0.40, *p* = 0.53). The highest DOC release rate was observed during the daytime in summer (17.5 ± 15.9 nmol C cm^−2^ h^−1^) while the lowest value was observed during nighttime in winter (−12.0 ± 14.8 nmol C cm^−2^ h^−1^). Additionally, DOC released during daytime in winter (7.3 ± 17.1 nmol C cm^−2^ h^−1^) was significantly higher than during the nighttime (Tukey HSD test, *p* < 0.05, Fig. 1; Table 1).

### Coral metabolism (calcification, photosynthesis, respiration)

**Figure 2:**
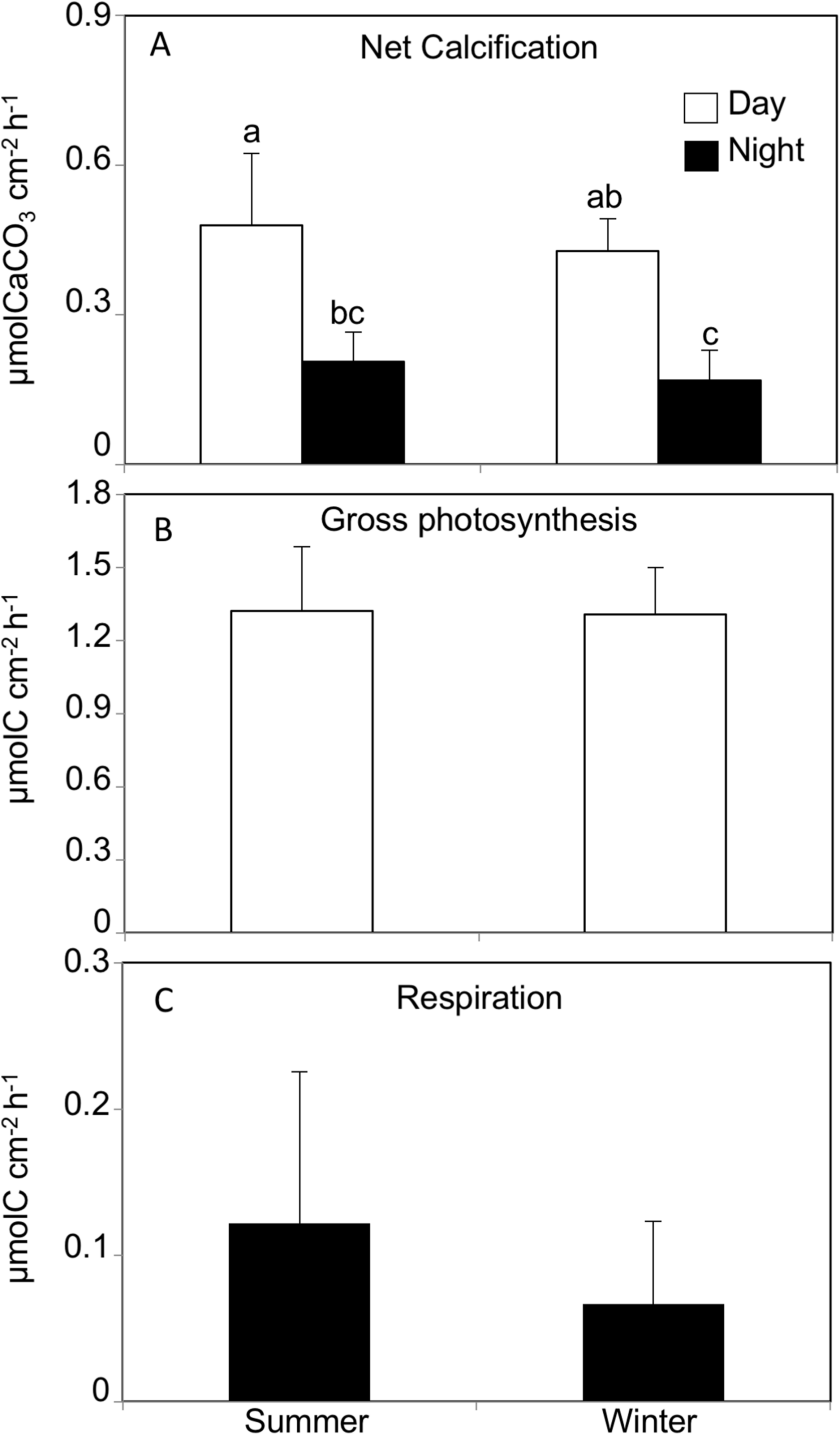
(A) Net calcification, (B) net photosynthesis and (C) respiration rates by *Acropora tennis* in summer and winter during day and night time. Values are mean and SD (*n* = 12). Bars with different small letters show significant differences among treatments (HSD Tukey–Krammer, *p* < 0.05).

The daytime calcification rate (Light G) was significantly higher than the nighttime calcification rate (Dark G) in both summer (Light G: 0.47 ± 0.14, Dark G: 0.20 ± 0.05 μmol C cm^−2^ h^−1^) and winter (Light G: 0.42 ± 0.06, Dark G: 0.16 ± 0.06 μmol C cm^−2^ h^−1^, REML, *F*_(1,40)_ = 180, *p* < 0.0001, Tukey HSD *p* < 0.05), while there was no significant difference between seasons (REML, *F*_(1,4)_ = 0.63, *p* = 0.47), and no interaction (REML, *F*_(1,40)_ = 1.18, *p* = 0.28, Fig. 2; Table 1).

The gross photosynthesis rate in corals incubated in summer and winter daytime averaged 1.32 ± 0.26 μmol C cm^−2^ h^−1^ and 1.30 ± 0.19 μmol C cm^−2^ h^−1^, respectively, and there was no significant difference between seasons (REML, *F*_(1,4)_ = 0.002, *p* = 0.98). Similarly, the respiration rates in corals incubated in summer and winter nighttime were 0.12 ± 0.10 μmol C cm^−2^ h^−1^ and 0.06 ± 0.05 μmol C cm^−2^ h^−1^, respectively, and there was no significant difference between seasons (Wilcoxon *p* = 0.20, Fig. 2).

## Discussion

The scleractinian coral *A. tenuis* released significantly more mucus as POM (POC and PON) and DOM (DOC) during the daytime. This result is consistent with previous studies by Crossland, Barnes & Borowitzka (1980) and Crossland (1987), demonstrating that POM release by the corals *A. variabilis* and *S. pistillata* was higher during the day. These results suggest that light intensity greatly influences mucus release in both particulate and dissolved forms. Crossland (1987) reported that the release of mucus was maximal in the afternoon and suggested that this could reflect the utilization of carbon fixed by photosynthetic activity of the symbiotic dinoflagellates. In this study, we also found that the C:N ratio of POM was significantly higher during the day (summer: 12.8 ± 5.7, winter: 12.0 ± 4.1) than the night (summer: 6.1 ± 2.5, winter: 2.2 ± 1.8) in both the summer and winter seasons. This result is in line with the assumption that the more energy-rich, nitrogen-poor products of photosynthesis are transferred to the host during the daytime (Marsh, 1970) and these are being used for mucus formation. Similarly, Dubinsky & Jokiel (1994) also indicated that higher amount of carbon is excreted as mucus with higher C:N ratios, under high light and low inorganic nutrient conditions. The reported C:N ratio of coral mucus showed a wide range of values from 4 to 16 (Table 2), while C:N ratio at winter nighttime of *A. tenuis* was lower than this range. As well, previously, a decrease of C:N ratio in aged mucus was observed in *Porites* spp. mucus sheets, which was interpreted to be due to “nitrogen-enrichment” by bacteria that absorb carbon but not nitrogen (Coffroth, 1990). However, taking into account that newly released POM from coral was evaluated in this study, this very low C:N ratio measured at winter nighttime could be an artifact. Nevertheless, the present results also suggest that both the quantity and quality of the mucus released from the corals exhibits diurnal change.

**Table 2:** Summary of previous data evaluating the release rate of particulate and dissolved matter of the coral *Acropora spp*.

Coral	Method	Stir	DOC (nmol cm^−2^ h^−1^)	POC (nmol cm^−2^ h^−1^)	DOC:POC	POC:PON	Temp (C°)	Light (μmol quanta m^−2^ s^−1^)	Place	TOC/NPC(%)	Reference
*Acropora tenuis*	Bottle	Yes	18	48	0.4	13	28.8	390	Okinawa Isalnd, Japan	6.9%	This study
	Bottle	Yes	7	25	0.3	12	20.1	355		1.6%	
*Acropora* sp	Bottle	No	33	24	1.4	9	29	400	Aqaba, Jordan	–	Naumann et al. (2010b)
	Bottle	No	0	8	0.0	5	21	216		–	
*Acropor nobilis*	in situ, plastic bag	No	220	101	2.2	4	28.5	Sunlight	Bidong Island, Malaysia		Nakajima et al. (2009)
*Acropora formosa*	Bottle	No	124	74	1.7	–	29.5	Sunlight	Tioman Island, Malaysia		Nakajima et al. (2010)
*Acropora variabilis*	Flow-through bottle	No	77	^**–**^	^**–**^	–	–	400–670	Aqaba, Islael	8%	Crossland (1987)
*Acropora pulchra*	Bottle	Yes	37	–	–	–	28.7	Sunlight	Ishigaki Island, Japan	5.4%	Tanaka et al. (2009)
*Acropora pulchra*	Bottle	Yes	31	62	0.5	16	28.7	Sunlight	Ishigaki Island, Japan	>10%	Tanaka et al. (2008)
*Acropora millepora*	Bottle	No	–	83	–	7.6	26–28	Sunlight	Heron Island, GBR		Wild, Woyt & Huettel (2005)
*Acropora aspera*	Bottle	No	–	58	–	–		Sunlight	Heron Island, GBR		Wild, Woyt & Huettel (2005)
*Acropora millepora*	Bottle	Yes	31	41	0.8	–	26.5	275	Fiji		Levas et al. (2015)
	Bottle	Yes	16	28	0.6	–	31	275			

**Note:**

Modified from the Figs. 2 and 3 from Nakajima & Tanaka (2014).

The POC and DOC release rates of *A. tenuis* were significantly higher in summer than winter, although no difference was found in PON release. Taking into account the fact that daytime duration during summer and winter at this study site (Okinawa Island) averages 14 and 11 h, respectively, the daily amount of POC and DOC can be roughly estimated to be 808 and 342 μmol C cm^−2^ d^−1^ for summer and 295 and −76 μmol C cm^−2^ d^−1^ for winter, respectively. This suggests that the total amount of carbon released per day as mucus is five times higher in summer compared to winter. When in situ DOC concentration was measured at a Caribbean reef, it was found that DOC concentrations were lower during seasons that showed lower light intensity, although correlation with light intensity and DOC release was only shown in benthic algae and not in corals (Mueller et al., 2014). When the mucus release rates of six different coral genera (*Acropora*, *Fungia*, *Stylophora*, *Goniastrea*, *Pocillopora* and *Millepora*) were measured through four different seasons, although only the POC release rate of *Acropora* corals in winter was significantly lower compared to other seasons, significant correlation was found with temperature and light intensity for POC and PON released from *Acropora* and *Fungia* corals (Naumann et al., 2010b). Since the net photosynthesis rate in this study was not significantly different between summer and winter, temperature could be one of the main factors that influenced the rate of mucus release from *A. tenuis* among seasons, in addition to light intensity. Since symbiotic dinoflagellate division rates are known to increase under lower light intensities and higher nutrient concentrations, such as found in winter (Hoegh-Guldberg, 1994), these conditions could result in a reduction in photosynthetic rates per dinoflagellate and in translocation of photosynthate from dinoflagellate to the host (Dubinsky & Jokiel, 1994). Such changes could be another explanation for the lower POC and DOC release rates in winter.

The POC and DOC release rates (POC: 25–48, DOC: 7–17 nmol cm^−2^ h^−1^) during summer from *A. tenuis* in the present study were close to the values reported by Tanaka et al. (2008) (*A. pulchra* POC: 62, DOC: 31 nmol cm^−2^ h^−1^) and Levas et al. (2015) (*A. millepora* POC: 28–50, DOC: 8–31 nmol cm^−2^ h^−1^) using similar methods (incubation chambers in the laboratory under artificial lightning and stirring). Additionally, the daytime DOC:POC values in the present study (summer: 0.5 ± 0.5, winter: 0.32 ± 0.98) are similar to the value reported for *A. pulchra* (0.5 ± 0.03) by Tanaka et al. (2008), reinforcing the higher release of mucus in particulate form than dissolved form by these corals. The DOC:POC value of *Acropora* ranged from 0.0 to 2.2, in agreement with previous studies (Table 2). However, POC and DOC release rates differ greatly among studies, among coral genera and even within the same genera. For *Acropora* spp., the reported values for POC and DOC range from 8 to 101, and 0 to 220 nmol cm^−2^ h^−1^, respectively (Crossland, 1987; Wild, Woyt & Huettel, 2005; Tanaka et al., 2008, 2009; Nakajima et al., 2009, 2010; Naumann et al., 2010b; Nakajima & Tanaka, 2014; Levas et al., 2015) (Table 2). The reason for this high variation has been proposed to be methodological differences, such as the method of mucus collection (air exposure, water jets, incubation chamber in laboratory or in field (Tanaka et al., 2009; Nakajima & Tanaka, 2014), lightning with artificial or natural light (Nakajima & Tanaka, 2014)), and species specificity (Naumann et al., 2010b). Additionally, although chamber incubation methods have been used in most studies to quantitatively evaluate the amount of mucus released by corals in water (Tanaka et al., 2008, 2009; Tanaka, Ogawa & Miyajima, 2010; Naumann et al., 2010b; Levas et al., 2015), stress by enclosing corals within a chamber may affect coral physiology and mucus release. Principally, water flow is known to strongly affect the physiology of corals (Patterson, Sebens & Olson, 1991), and for example, all net photosynthesis, respiration and calcification rates in the coral *A. formosa* were reported to decrease by about 20% in unstirred conditions compared to stirred conditions (Dennison & Barnes, 1988). Here we stirred the water in the chambers to create continuous flow during the experiment. Flow speeds on the reef can vary greatly depending on prevailing conditions; normal flow at three m depth on the forereef has been estimated to range from 1 to 40 cm s^−1^ (Carpenter, Hackney & Adey, 1991). Hence the flow rate used in the present study (average 3.4 cm s^−1^) can be taken to be equivalent to calm conditions. However, it is also worth noting that when primary production of coral reef algae was compared between a chamber under an oscillatory flow regime and vortex flow created by stirring, algae production was 21% higher under oscillation flow (Carpenter, Hackney & Adey, 1991). Additionally, Naumann et al. (2010b) have pointed out that DOC:POC values could be affected by stirring of seawater. Although limitations of chamber incubation methods should be acknowledged, for further understanding of mucus production under different environmental conditions we feel it is worthwhile to mitigate the stress of chambers to subject corals by minimizing periods of incubation, flushing out seawater within the chamber with each incubation, and decreasing the coral volume relative to the water volume within chambers.

Possible rapid degradation of DOM released from corals due to bacterial growth could result in the underestimation of coral mucus release rates. Ferrier-Pagès et al. (1998) demonstrated substantial DOC uptake by bacteria associated with the coral *Galaxea fascicularis.* Wild et al. (2004) reported significantly higher bacterial numbers, by about 100-fold, in coral mucus compared to surrounding seawater. In addition, when coral mucus was incubated within a chamber, the concentration of DOC was found to significantly decrease followed by an increase in bacterial abundance within a few hours of incubation (Nakajima et al., 2009, 2015). Additionally, Nakajima et al. (2017) also demonstrated that the coral-derived DOM not only increased bacterial abundance but also heterotrophic nanoflagellate abundance, suggesting that coral mucus can be an efficient carbon source that fuel the microbial loop. Since in this study the mean DOC release rate during the night in winter showed negative values, one suggestion could be DOC uptake during the 3 h incubation by bacteria. Similar DOC uptake has been reported for the corals *Fungia*, *Stylophora* and *Pocillopora* during 4 h incubation across all four seasons, while uptake was not evident for other corals such as *Goniastrea* and *Millepora* (Naumann et al., 2010b). Additionally, *Acropora* was found to show DOC uptake only during the night (Naumann et al., 2010b). Another suggestion could be DOM uptake by the coral itself. DOM uptake by the coral *G. fascicularis* was reported to increase at lower temperature (Al-Moghrabi, Allemand & Jaubert, 1993), and has been proposed to increase in winter to compensate for lower productivity due to lower light intensity (Davies, 1991). Levas et al. (2016) also reported that the corals *Porites divaricata* and *Orbicella faveolata* released DOC during daytime, and took up DOC during nighttime, and similarly Haas et al. (2010) hypothesized that corals can take up DOC and utilize as a carbon source.

In this study, there were no significant difference in photosynthesis rates between seasons. Crossland (1981, 1984) also reported that the photosynthesis rate of the coral *A. formosa* incubated under natural light conditions in the Abrolhos Island, Western Australia did not show significant seasonal changes. Meanwhile, the net photosynthesis rates of the corals *Porites compressa* and *Montipora verucosa* studied at a community scale were about two times higher in summer compared to winter (Langdon & Atkinson, 2005). Photosynthesis rates are known to show a hyperbolic tangent in relation with light intensity (Chalker, Dunlap & Oliver, 1983), while under saturated light conditions, photosynthesis rates increase linearly with temperature (Coles & Jokiel, 1977). Symbiotic dinoflagellate density and chlorophyll pigment contents have been reported to fluctuate among seasons, showing significant declines with rising seawater temperature and light intensity (Brown et al., 1999; Fitt et al., 2000; Stimson, 1997). Brown et al. (1999) also reported that symbiotic dinoflagellate density and chlorophyll *a* and *c*_2_ concentrations were higher during the rainy season under lower light and temperature than during the dry season. Additionally, Xu et al. (2016) reported that the photosynthesis efficiency (Fv Fm^−1^) of photosynthesis II was significantly higher in winter compared to summer in five coral species. Warner et al. (2002) reported that symbiotic dinoflagellate density and Fv Fm^−1^ of the coral coral *Montastrea annularis* showed significant seasonal fluctuations, with the highest symbiotic dinoflagellate density and Fv Fm^−1^ values occurring in winter. Therefore, the photo-acclimation responses by symbiotic dinoflagellates and corals to lower light intensity could be one factor leading to diminished differences in photosynthesis rates between summer and winter coral in this study. Additionally, it should also be noted that the light intensities used during the incubation were slightly lower for summer and higher for winter compared to the mean values measured outdoors, which may have masked any differences in photosynthesis rates between summer and winter corals in this study. Based on the measured net photosynthesis rates in this study, the daily net carbon fixation rates in summer and winter by *A. tenuis* can be roughly estimated to be 16.6 μmol C cm^−2^ d^−1^ and 13.6 μmol C cm^−2^, respectively. These values are consistent with previous work that estimated daily net carbon fixation of 23 μmol C cm^−2^ for *A. acuminata* (Crossland, Barnes & Borowitzka, 1980) and 10 μmol C cm^−2^ for *A. pulchra* (Tanaka et al., 2007). Taking into account the estimated daily release rates of POC (S: 807, W: 294 nmol C cm^−2^ d^−1^) and DOC (S: 286, W: −75 nmol C cm^−2^ d^−1^) mucus was calculated to account for 6.5% (DOC: 1.7%, POC: 4.8%) and 1.6% (DOC: −0.5%: negative value due to DOC uptake, POC: 2.1%) of the net carbon fixation in summer and winter, respectively. From these results, it can be suggested that the relative rates of DOC and total organic carbon (TOC) release to coral carbon fixation can change seasonally. With the observed change of daily DOC release in summer to DOC uptake in winter seasons, it can be suggested that the coral *A. tenuis* has the ability to change the carbon source and energy allocation among seasons. The calcification and respiration rates measured for *A. tenuis* in this study did not significantly change between seasons, which contradicts previous studies showing that calcification and respiration are positively correlated with temperature (Crossland, 1984; Coles & Jokiel, 1977; Marshall & Clode, 2004). If this is the case for most corals, the relative energy consumption used for mucus release could become significant principally in the summer season.

The value for the ratio of mucus per net photosynthesis estimated in this study is consistent with the value of about 10% reported by Tanaka et al. (2008). Meanwhile, the present value was lower than values estimated in some previous studies; 40% (Crossland, 1987), 49% (Davies, 1984), 10–21% (Crossland, 1987) and 14% (Ferrier-Pagès et al., 1998). It is known that amount of mucus released by corals can greatly increase under stressed conditions such as sedimentation (Reigl & Branch, 1995), air exposure (Daumas & Thomassin, 1977) and pollutants (Meikle, Richards & Yellowlees, 1988). Therefore, although the percentage of C released as mucus compared to net C fixation in coral (i.e., 1.6–6.5%) was estimated to be less than previously estimated (40–50%), mucus production may vary with environmental changes and become a potentially significant component of the coral energy budget.

## Conclusions

The daily rates of mucus released by the coral *A. tenuis* and the percentage of mucus compared to net carbon fixation were found to be significantly higher in summer compared to winter. Additionally, the release rate of mucus, and the POC:PON ratios in the mucus, were significantly higher during the day compared to the night. These results demonstrate diurnal and seasonal changes in both the quantity and quality of mucus released from this coral. Taking into account the fact that the mucus of corals is used as a food source by other reef macro-organisms, and also serve as energy source for micro-organisms, the observed changes in mucus release could influence the seasonal dynamics of organic carbon and nitrogen cycling.

## Supplemental Information

10.7717/peerj.5728/supp-1 Supplemental Information 1 Raw data for Figure 1. The data for POC, PON and DOC release by corals incubated at day and night time in summer and winter.

10.7717/peerj.5728/supp-2 Supplemental Information 2 Raw data for Figure 2. Net calcification, gross photosynthesis and respiration rate of corals at summer and winter.

## Additional Information and Declarations

### Field Study Permissions

The following information was supplied relating to field study approvals (i.e., approving body and any reference numbers): All corals were collected under permission from Okinawa Prefecture (#24–65).

### Data Availability

The following information was supplied regarding data availability: The raw data are provided in the Supplemental Files.
